# *Pseudomonas fluorescens* F113 type VI secretion systems mediate bacterial killing and adaption to the rhizosphere microbiome

**DOI:** 10.1038/s41598-021-85218-1

**Published:** 2021-03-11

**Authors:** David Durán, Patricia Bernal, David Vazquez-Arias, Esther Blanco-Romero, Daniel Garrido-Sanz, Miguel Redondo-Nieto, Rafael Rivilla, Marta Martín

**Affiliations:** 1 Departamento de Biología, Facultad de Ciencias, Universidad Autónoma de Madrid, Darwin, 2, 28049 Madrid, Spain; 2 Departamento de Microbiología, Facultad de Biología, Universidad de Sevilla, Avenida de la Reina Mercedes, 6, 41012 Sevilla, Spain

**Keywords:** Microbiology, Bacteria, Bacterial secretion

## Abstract

The genome of *Pseudomonas fluorescens* F113, a model rhizobacterium and a plant growth-promoting agent, encodes three putative type VI secretion systems (T6SSs); F1-, F2- and F3-T6SS. Bioinformatic analysis of the F113 T6SSs has revealed that they belong to group 3, group 1.1, and group 4a, respectively, similar to those previously described in *Pseudomonas aeruginosa*. In addition, in silico analyses allowed us to identify genes encoding a total of five orphan VgrG proteins and eight putative effectors (Tfe), some with their cognate immunity protein (Tfi) pairs. Genes encoding Tfe and Tfi are found in the proximity of *P. fluorescens* F113 *vgrG*, *hcp*, *eagR* and *tap* genes. RNA-Seq analyses in liquid culture and rhizosphere have revealed that F1- and F3-T6SS are expressed under all conditions, indicating that they are active systems, while F2-T6SS did not show any relevant expression under the tested conditions. The analysis of structural mutants in the three T6SSs has shown that the active F1- and F3-T6SSs are involved in interbacterial killing while F2 is not active in these conditions and its role is still unknown.. A rhizosphere colonization analysis of the double mutant affected in the F1- and F3-T6SS clusters showed that the double mutant was severely impaired in persistence in the rhizosphere microbiome, revealing the importance of these two systems for rhizosphere adaption.

## Introduction

The Type six secretion system (T6SS) was originally described in *Vibrio cholerae*^1^ and *Pseudomonas aeruginosa*^2^ as a proteinaceous nanomachine that translocates specific proteins directly into target cells^3^. T6SSs are present in more than 25% of gram-negative bacteria, mostly confined to the phylum *Proteobacteria*^4,5^ and many of them encoding more than one T6SS in their genome^6,7^. Initially, the T6SS was described as a classical virulence factor against eukaryotic cells, including humans, other animals^3,8,9^ and plants^10–13^. However, soon after, it was shown that its relevance resides mainly within its anti-prokaryotic activity^14–17^.

The core genes of the T6SSs are located in genomic clusters commonly comprised of 13 to 15 genes, which encode the structural proteins of the system with well-conserved functions^7,18–20^. A comprehensive phylogenetic study of T6SS clusters carried out by^7^ cladded these systems into five main phylogenetic groups (1–5). A subsequent study in the *Pseudomonas* genus classified the T6SS loci into six different phylogenetic groups (1.1, 1.2, 2, 3, 4a, and 4b) due to the division in two subgroups of clades 1 and 4 and the absence of clade 5^21^.

The encoded-core proteins are named Tss (type six secretion)^18^ and include the structural components that form the membrane complex, the baseplate, and the tail. The assemblage of the system starts with the anchoring of the membrane complex (TssJLM) to the membrane and its interaction with a hexameric ring of TssA proteins^22,23^. The TssA ring mediates the interaction between the membrane complex and the baseplate (TssEFGK), where the tip of the system, a trimer of VgrG proteins topped with a PAAR domain, sits. From the distal end of the baseplate, TssA ring primes the polymerisation of the tail, starting with the Hcp inner tube and continuing with the surrounded sheath (TssBC), and it moves down capping the tail until it reaches the other side of the cell^22^^.^^24^. Once the system has completed the assembling and upon an unknown signal, the sheath contracts ejecting the Hcp-VgrG effector-loaded structure out of the producing cell and inside the target cell^25–27^. The components of the contractile sheath (TssBC) are recycled by an ATPase named ClpV^28–32^.

Besides the structural proteins, these clusters can encode accessory proteins named Tag (type VI accessories genes) such as TagABFJLPRSTQ involved in regulation or other mechanistic aspects of the T6SSs^7,24,33–35^. Moreover, genes encoding T6SS effectors and their cognate immunity proteins are commonly linked to *hcp* and/or *vgrG* genes within T6SS clusters^36–38^. Most T6SS effectors are anti-bacterial toxins but some anti-eukaryotic effectors have been also described in bacterial pathogens such as *V. cholerae* and *P. aeruginosa*^29,39,40^; interestingly, T6SS antifungal effectors have also been found in *Serratia marcescens* and proved to inhibit the growth of pathogenic *Candida* species^17^. Additionally, genes encoding immunity proteins are found invariably adjacent to genes encoding their cognate effectors. Immunity proteins protect T6SS-producing cells from self-intoxication and the attack of T6SSs from sister cells^14,41–44^. Genes encoding orphan VgrG proteins, not genetically linked to any T6SS structural cluster, have also been described for a large number of T6SS-containing bacteria^21,45,46^. These orphan VgrG-encoding genes are frequently linked to genes encoding chaperone proteins and effector-immunity pairs (EI pairs)^4,15,42,47,48^.

Within pseudomonads, T6SSs have been extensively studied in *P. aeruginosa*^14,49–54^ and more recently in *Pseudomonas putida*, where it has been shown relevant for its activity against phytopathogens^24,38^. In other *Pseudomonas* species, T6SSs have been implicated in the production of the siderophore pyoverdine, i.e., *Pseudomonas taiwanensis*^55^, and bacterial colony invasion, i.e., *Pseudomonas chlororaphis*^56^. However, limited information is available for other pseudomonads, especially within the species belonging to the *P. fluorescens* complex. The T6SS of *Pseudomonas fluorescens* MFE01 has been involved in biofilm formation^57^, whereas *P. fluorescens* Arp29 T6SSs are expressed in the rhizosphere environment but their functions are unknown^58^. In *Pseudomonas protegens*, a functional T6SS has been found^59^ and some antibacterial toxins have been characterized^60,61^. The model organism of this study, *Pseudomonas fluorescens* F113, is a prototypical rhizobacterium isolated from the sugar-beet rhizosphere^62^. The genomic sequence of *P. fluorescens* F113 has been described^63,64^, revealing a large set of essential rhizosphere-adaptative and plant growth-promotion traits, including the presence of three T6SSs. This work aims to analyse the phylogeny of the T6SSs in the *P. fluorescens* species complex and to characterize the three T6SSs present in *P. fluorescens* F113 together with their effectors.

## Results and discussion

### Distribution of T6SSs in the *Pseudomonas fluorescens* species complex

**Figure 1:**
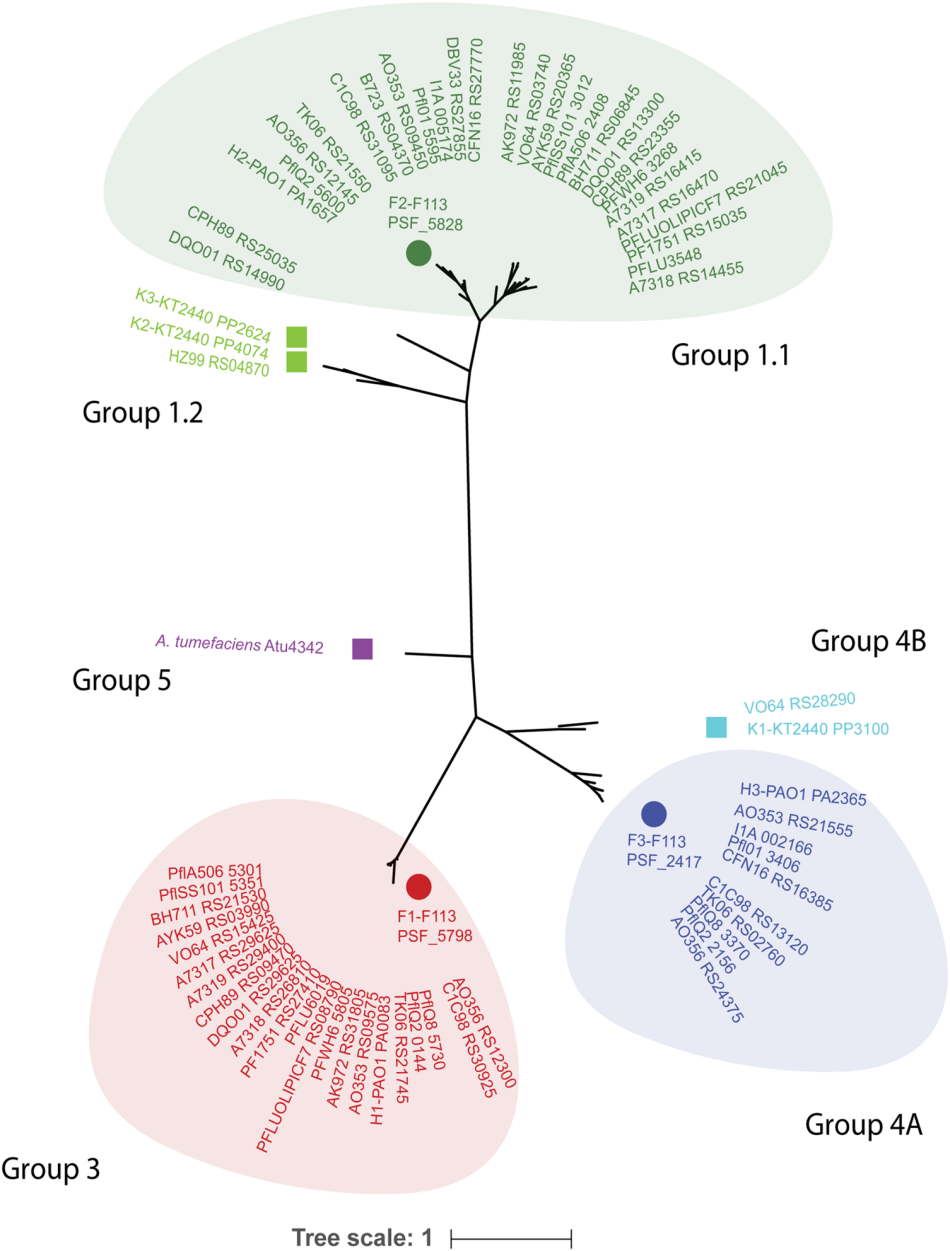
Phylogenetic relationship of T6SS clusters in *P. fluorescens* species. The Maximum likelihood tree with 1000 bootstrap replicates was built with Mega X with the core component protein TssB. The T6SS clusters from *P. fluorescens* F113 are divided into three distinguishable groups; 1.1, 3, 4a. The *A. tumefaciens* TssB was used as an outgroup (group 5).

**Figure 2:**
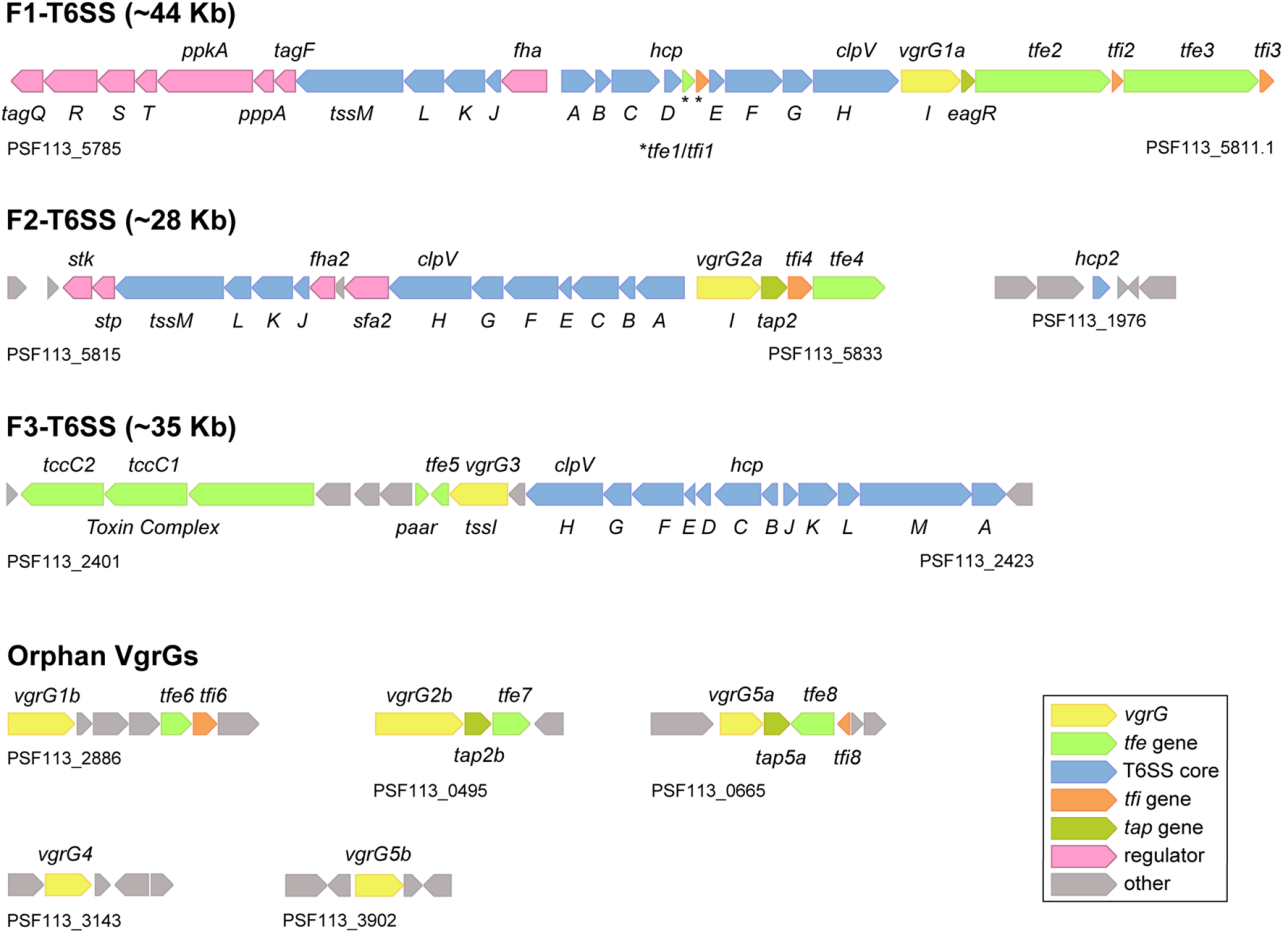
Genetic organization of T6SS clusters in *P. fluorescens* F113. Schematic representation of the F1-, F2-, F3-T6SS clusters and the *vgrG* and *hcp* orphan clusters. The colour code of the genes indicates their predicted role according to the colour legend shown. The core genes *tssA*-*M* are indicated by a letter. Genes are represented as blocked arrows showing the direction of their transcription. The genes are to scale.

In silico analyses of the genomes of 134 strains belonging to the *P. fluorescens* species complex revealed that only 20% of them encode T6SS clusters (Table S3). The number of T6SS clusters in a single strain fluctuated from zero in *P. fluorescens* UK4 to three in *P. fluorescens* F113, and most strains contained two or three clusters (Table S3). Overall, we identified sixty-two complete T6SS gene clusters distributed mostly in three main phylogenetic clades. We referred to these three groups as 1.1, 3, and 4A (Fig. 1) following the previous nomenclature^7,21^. The distribution of the clusters in these three groups is the same as in *P. aeruginosa*^2,11^. No clusters were found corresponding to group 5, typical of *Agrobacterium* spp. and very few belonged to clusters 1.2 and 4B as in *P. putida*^38^. Each of these groups contains distinguishable genetic architecture and features (Fig. 2), as described in the next section.

### *Pseudomonas fluorescens* F113 contains three putative T6SSs

An extensive genomic analysis of the strain F113 using bioinformatics approaches (e.g., BLASTP, SMART, or Phyre-version 2) allowed us to identify a large number of T6SS-related ORFs (Tables S4-S7). Most of the genes are clustered in three genomic regions that we have named F1-, F2- and F3-T6SS (Fig. 2, Tables S4-S6). We identified a total of three *hcp* and eight *vgrG* genes,one of the *hcp* and six of the vgrG are orphan genes that were found scattered on the chromosome and not within the main T6SS clusters (Fig. 2 and Table S7). Phylogenetic analysis revealed that similarly to *P. aeruginosa* PAO1, the F1-T6SS belongs to phylogenetic group 3, F2-T6SS to group 1.1 and the F3-T6SS to group 4A (Fig. 1).

Each of the F113 T6SS clusters (F1-, F2- and F3-T6SS) consists of two divergently transcribed gene clusters that contain the 13 predicted core-genes (*tssABCDEFGHIJKLM*) (Fig. 2 and Tables S4-S6) and genes putatively encoding accessory components as described below.

The large F1 cluster (44 Kb) contains a set of genes putatively encoding regulatory proteins previously described in *P. aeruginosa*: *fha*, *tagF*, *pppA*, *ppkA*, and *tagRSTQ.* Interestingly, it does not contain the gene that encodes the accessory component TagJ (Fig. 2) present in *P. aeruginosa* H1-T6SS^24,65^ and conserved in other clusters from the same phylogenetic group (Group 3). In those clusters, *tagJ* is found located downstream *hcp* and upstream *tssE,* and it has been recently described as an accessory component that stabilizes the sheath from the baseplate^24^*.* Instead, in this location, *P. fluorescens,* contains the orthologues of *tae4*-*tai4* from *Salmonella typhimurium,* which encode an EI pair (see below). A gene encoding a DUF1795 domain-containing protein is found downstream *vgrG1a* within the F1-cluster. The product of this gene is also known as an EagR chaperone for a downstream Rhs type effector first identified in *Serratia marcescens*^40^. Two Rhs-type effectors are encoded downstream *vgrG1a* and the *eagR* chaperone in F113 (Fig. 2) and are described in the following section.

The F2-T6SS is a shorter cluster (around 29 Kb) with four genes putatively encoding regulatory proteins previously defined in *P. aeruginosa*^66^ (*stp*, *stk*, *fha2* and *sfa2*). All the core components are present within the cluster except for *hcp2* (PSF113_1976) that is found in a different genomic region. A gene encoding a DUF4123 domain-containing protein chaperone^37,48^ (Tap2) is found downstream *vgrG2a* followed by a hypothetical protein and a lipase, that are likely to correspond to an immunity-toxin pair (Fig. 2 and Table S5).

The F3-T6SS cluster, with an intermediate size of 35 Kb, does not contain genes encoding regulatory proteins (Fig. 2 and Table S6), as opposed to *P. aeruginosa*, which contains *sfa3*, a gene encoding a putative sigma-54 transcriptional regulator enhancer. Interestingly, further down the T6SS cluster and three no-T6SS genes, we identified genes putatively encoding a Toxin Complex (TC) composed of one TcdB and two TccC subunits. The absence of the TcdA subunit, which forms an injection-like structure, has been observed in some strains^67^ and it opens the possibility for this complex to be secreted through the T6SS.

A total of eight *vgrG* genes are found in the chromosome of *P. fluorescens* F113. Three of these *vgrG* genes are within the F1-, F2- and F3-T6SS, whereas the other five genes are scattered over the chromosome (Table S7). A phylogenetic study of the eight VgrG proteins shows that VgrG1a and 1b clustered in the same phylogenetic group as *P. aeruginosa* VgrG1 proteins. On the other hand, *P. fluorescens* VgrG2a/b, VgrG4, and VGrG5a/b are closely related among them and cluster with *P. aeruginosa* VgrG4, 5 and 6 (Figure S1).

### Identification of eight putative type 6 effectors in *P. fluorescens* F113

**Figure 3:**
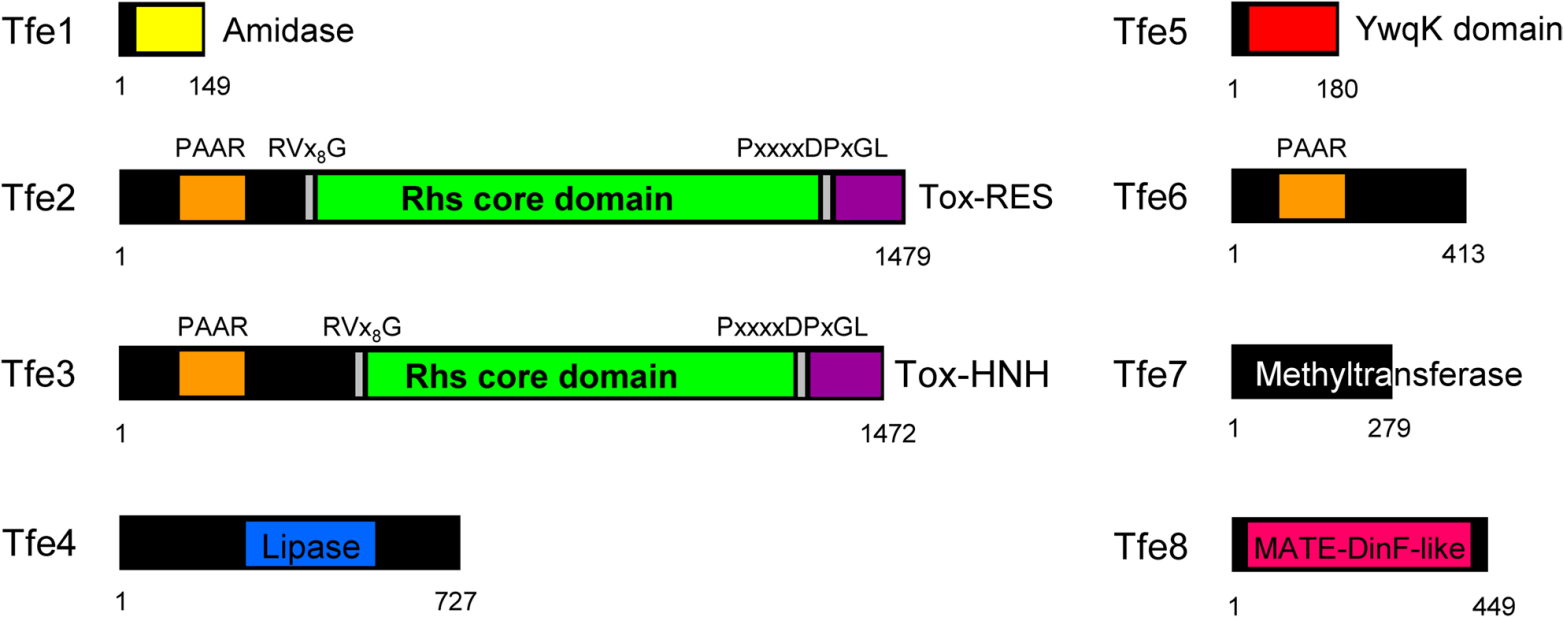
Domain composition of T6SS-related effectors present in *P. fluorescens* F113. The domain organization of the putative effectors is shown, with amidase domain in yellow, PAAR motifs indicated in orange, RVxxxxxxxxG and PxxxxDPxGL motifs in grey, Rhs domains in green, HNH nuclease motifs (Tox-HNH and Tox-SHH) in purple, YwqK domain in red and MATE domain in pink. Structural-based homology prediction was determined using the Protein Homology/analogy Recognition Engine (Phyre) server.

As stated before, genes encoding T6SS effector and their cognate immunity proteins are frequently found genetically associated to *hcp* and *vgrG* genes and in some cases to genes encoding T6SS chaperones or adaptors (i.e., EagR and Tap/Tec proteins)^15,36,37,48,68^. We have performed an in silico analysis that allowed us to identify a total of eight putative EI pairs in the proximity of F113 *vgrG*, *hcp, eagR*, and *tap* genes (Fig. 2 and Tables S4-S7). These EI pairs have been named Tfe and Tfi standing for Type six F113 effector and immunity, respectively (Figs. 2, 3 and Tables S4-S7).

Within the F1 cluster and downstream *hcp1*, we have identified the EI pair *tfe1-tfi1.* Tfe1 is orthologue to *Salmonella typhimurium* Tae4. Tae4 is a type VI amidase effector, which degrades the peptidoglycan of the cell wall and whose immunity protein is Tai4^43^. Furthermore, a gene encoding an EagR chaperone (*eagR1*) is found downstream *vgrG1a* in the F1-cluster and followed by two genes (*tfe2* and *tfe3*) encoding Rhs effectors in a row. These effectors both have the same domain architecture: an N-terminal PAAR domain^69,70^, an intermediate conserved Rhs domain^71^ confined by specific RVxxxxxxxxG and PxxxxDPxGL motifs, and a C-terminal region encoding the toxic domain. The toxic domain of Tfe2 is a putative RES domain, whose orthologue in *P. putida* is known to cause depletion of intracellular NAD^+^ levels leading to inhibition of cell growth^72^ (Fig. 3). In parallel, the C-terminal domain of Tfe3 carries a putative nuclease of the HNH/ENDO VII superfamily with a conserved WHH domain. *tfe2* and *tfe3* putative cognate immunity pairs, named *tfi2* and *tfi3,* are found immediately downstream of the genes encoding their effectors and have 92 and 136 amino acids, respectively (Table S4 and Figs. 2, 3). Interestingly, *tfi3* is a newly identified ORF in the *P. fluorescens* F113 genome and we have named the locus PSF113_5811.1 to indicate that the locus is located downstream PSF113_5811 (Table S4 and Fig. 2). Whereas Tfi2 immunity protein has no homologues or recognizable features, Tfi3 belongs to the Smi1/Knr4 family, which has been previously related to immunity proteins of polymorphic toxins^73^.

Genes encoding putative effectors were also found within clusters F2 and F3, downstream *vgrG2a* and *vgrG3 *respectively and downstream orphan *vgrG* genes including *vgrG1b*, *vgrG2b* and *vgrG5a* (Fig. 2). The *tfe4, tfe7*, and *tfe8* genes within the F2 and *vgrG2b* and *vgrG5a* operons, respectively, are genetically associated with genes encoding Tap chaperones (*tap2*, *tap2b,* and *tap5a*) (Fig. 2). Tfe4 is a lipase from class 3, putatively targeting eukaryotic and prokaryotic membranes by hydrolysing long-chain acyl-triglycerides from lipids into di- and monoglycerides, glycerol, and free fatty acids. Tfi4 is the cognate immunity protein of Tfe4 and it does not contain any known function or recognisable domain (Table S5). Tfe7 is a homologue of a ribosomal RNA large subunit methyltransferase D required for the full methylation of 23S ribosomal RNA. Interestingly, no gene encoding an immunity pair can be found linked to this effector gene, indicating that it might not be necessary for the producer strain. This effector could replace a functional methyltransferase and only be toxic in those strains where this enzyme is necessary for bacterial viability. A similar case has been described in *P. aeruginosa* PAO1, in which the effector Tse8 is not linked to an immunity pair and replaces a functional component of the transamidosome complex only present in some strains^74^. Tfe8 may represent a new type of effector identified for the first time in this work and contains a MatE domain. None of these three Tap-linked effectors contains a conserved N-terminal MIX motif considered a marker for T6SS effectors^75^ and frequently found in Tap-associated effectors^38^.

Gene *tfe5* is located downstream *vgrG3* and linked to a small PAAR-encoding gene. Tfe5 is a homologue of the TseF toxin from *P. aeruginosa*, an iron scavenging effector that interacts with PQS vesicles to help bacteria grow under very limiting conditions^76^. A small gene located between *clpv3* and *vgrG3* encodes a protein of 188 amino acids with no recognizable features but has a C-terminal domain of 50 amino acids that resembles a coronavirus RNA-binding domain according to a Phyre prediction (confidence: 80.5). The *tfe6* is linked to the orphan *VgrG1b* cluster and has a similar genetic architecture to *P. aeruginosa* PAO1 VgrG1b cluster^53^. In PAO1 cluster, PA0095, PA0096, PA0097, PA0098, PA0099, PA0100, and PA0101 encode VgrG1b, an OB-fold, an immunoglobulin-like and a thiolase-like protein, a PAAR protein with a C-terminal cytotoxic domain, an immunity protein, and a heat repeat-containing protein respectively (Table S7). *Pseudomonas fluorescens* F113 *vgrG1b* cluster (PSF113_2885-PSF113_2890) only differs from the PAO1 one in the sequence of the toxic domain and the immunity pair, a common characteristic of this genetic island previously described by^53^. The specific function of Tfe6 and the mechanistic of the cognate immunity pair Tfi7 remains unknown.

No putative effector-encoding genes were identified linked to *hcp2*, *vgrG4,* and *vgr5b* clusters and we hypothesised that they could be found somewhere else in the chromosome as orphan T6SS effectors as described before in other T6SS clusters, including *Vibrio proteolyticus*^77^ among others.

In summary, we identified eight putative T6SS effectors in the F113 genome. Three of them, Tfe2, Tfe3, and Tfe6, contain an N-terminal PAAR-domain (Figs. 2, 3) and are considered “specialised” effectors, whether the others that are not fused to any T6SS component are considered “cargo” effectors.

### F1- and F3-T6SSs genes are expressed in *P. fluorescens* F113

**Figure 4:**
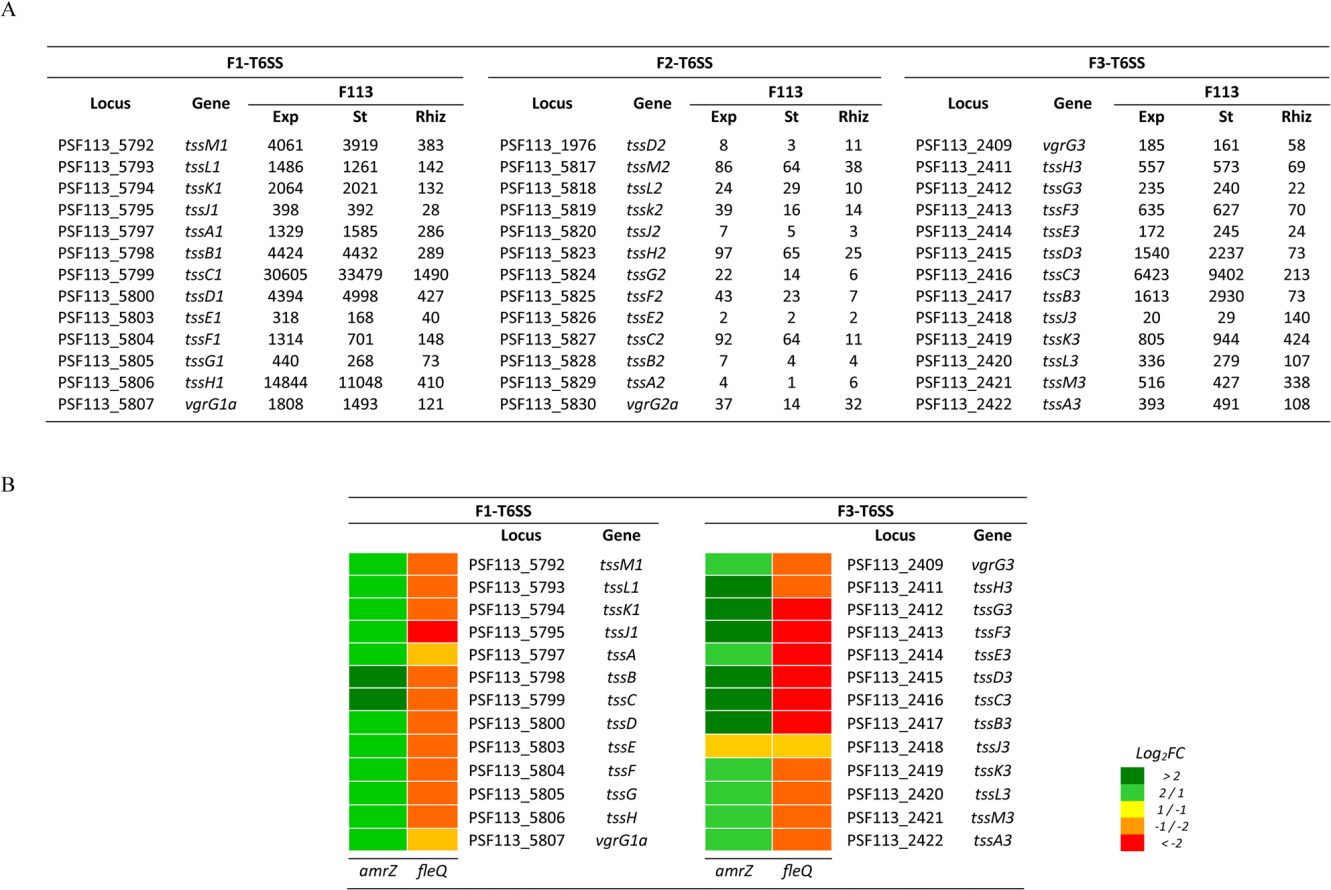
Expression of the *P. fluorescens* F113 T6SS genes. A.- RNA-seq analysis of the structural and *vgrG* T6SSs genes under the three different conditions tested. Gene expression values represent read counts normalized following the median of ratios method. Exp. culture in exponential growth phase (OD_600_ = 0.6); St. culture in stationary growth phase (OD_600_ = 1.2). Rhiz. Bacteria recovered from rhizosphere. B.- Heatmap representation of F1- and F3-T6SS gene expression in the *amrZ* and *fleQ* mutants background compared with wild type strain after rhizosphere colonization. *Pseudomonas fluorescens* F113 annotated genes with a log_2_FC (mutant/wild-type) ≤  − 1/ ≥ 1 are represented.

In order to determine the expression of genes encoding the components of the three T6SSs in *P. fluorescens* F113, we used a new dataset from an RNA-Seq study of F113 and derivatives grown under different culture conditions and in the alfalfa (*Medicago sativa*) rhizosphere. For this analysis, we chose the structural genes from each of the three systems (*tssABCDEFGHIJKLM*). As shown in Fig. 4A, F1- and F3- T6SS cluster genes were significantly expressed under all tested conditions: growing in liquid cultures of Minimal Sucrose-Asparagine (SA) at exponential and stationary phases and colonising the alfalfa rhizosphere. On the contrary, F2-T6SS genes showed little, if any, activity under the same conditions. Expression of F1-T6SS genes was similar in all culture conditions regardless of the growth phase analysed, and these genes were also expressed during rhizosphere colonization, suggesting that the F1-T6SS is constitutively expressed in F113. The expression of F3-T6SS genes is lower than that of the F1-T6SS genes in all conditions. This situation is similar to *P. putida*, which contains three clusters named K1-, K2- and K3-T6SS, that do not belong to the same phylogenetic groups of *P. fluorescens* F113. In *P. putida*, the K1-T6SS is constitutively expressed under laboratory conditions and is active in vitro and *in planta* assays^38^; however, no activity has been reported for K2- and K3-T6SS clusters to date. Conversely, in the case of *P. aeruginosa* that harbours the same groups as F113 and share regulatory components within the T6SS clusters, T6SS genes are not constitutively expressed and all of them are tightly regulated at transcriptional, post-transcriptional, and post-translational levels^11,78–80^. In PAO1, the three systems have the common regulator Rsm^52^ and the widely studied H1-T6SS is known to also be regulated by many other factors including RetS, GacA/S, TagF, PpkA/PppA, TagQRST, and FHA^2,33,34,80–82^.

To study the regulation of T6SSs during the process of rhizosphere colonization, we also analysed the T6SSs genes with differential expression in RNA-Seq data from the *P. fluorescens* F113 *amrZ* and *fleQ* mutants compared to the wild-type strain grown in the rhizosphere of alfalfa. AmrZ and FleQ are two transcription factors (TFs) crucial during competitive rhizosphere colonization in this bacterium^83–87^. AmrZ is a global regulator member of the AraC family of TFs that can act as a global and bi-functional regulator of gene expression in pseudomonads^88^ and it has been previously linked to the control of T6SS in *P. aeruginosa*^52^. FleQ belongs to the NtrC/NifA family and has been related to the regulation of T6SS and c-di-GMP metabolism in *P. putida*^89^. The differential gene expression analyses shown in Fig. 4B has revealed that in the rhizospheric environment, AmrZ functions as a negative transcriptional regulator of F1- and F3-T6SS, while FleQ acts as a positive regulator. This antagonist role of both transcriptional regulators fits with the proposed model for the AmrZ/FleQ hub, which has been proposed in F113 to act as an oscillator with opposing effects in gene expression, in order to integrate the bacterial responses to the environment^83^. In addition to the *vrgG* genes related to the F1- and F3-T6SS, most orphan *vrgG* genes are also negatively regulated by AmrZ under the tested conditions. However, FleQ can act both as a positive and negative regulator of certain *vgrG* genes. These results show that both AmrZ and FleQ regulate T6SSs in F113, as previously shown for AmrZ in *P. aeruginosa*^52^ and FleQ in *P. putida*^89^.

### *P. fluorescens* F113 F1- and F3-T6SS are implicated in bacterial killing

T6SS is a critical element in the antibacterial activity of some strains due to the injection of T6SS toxins into competitor target cells^5,7,30,42^. Therefore, to analyse the role of T6SS in *P. fluorescens* F113 in inter-bacterial competition, we performed bacterial killing assays as previously described^38^. In these assays, *P. fluorescens* F113 and its isogenic mutants were used as predators, and *Escherichia coli* harbouring a pK18*mobsacB* plasmid, containing the *lacZ* gene that confers blue colour to the colony in the presence of X-gal, was used as prey. Bacteria were mixed in a 1:1 ratio (predator:prey), co-cultured for 5 h and a sample of each mix was grown on selective plates for 48 h. Additionally, serial dilutions of the different assays were plated on selective media for predator and prey quantification.

**Figure 5:**
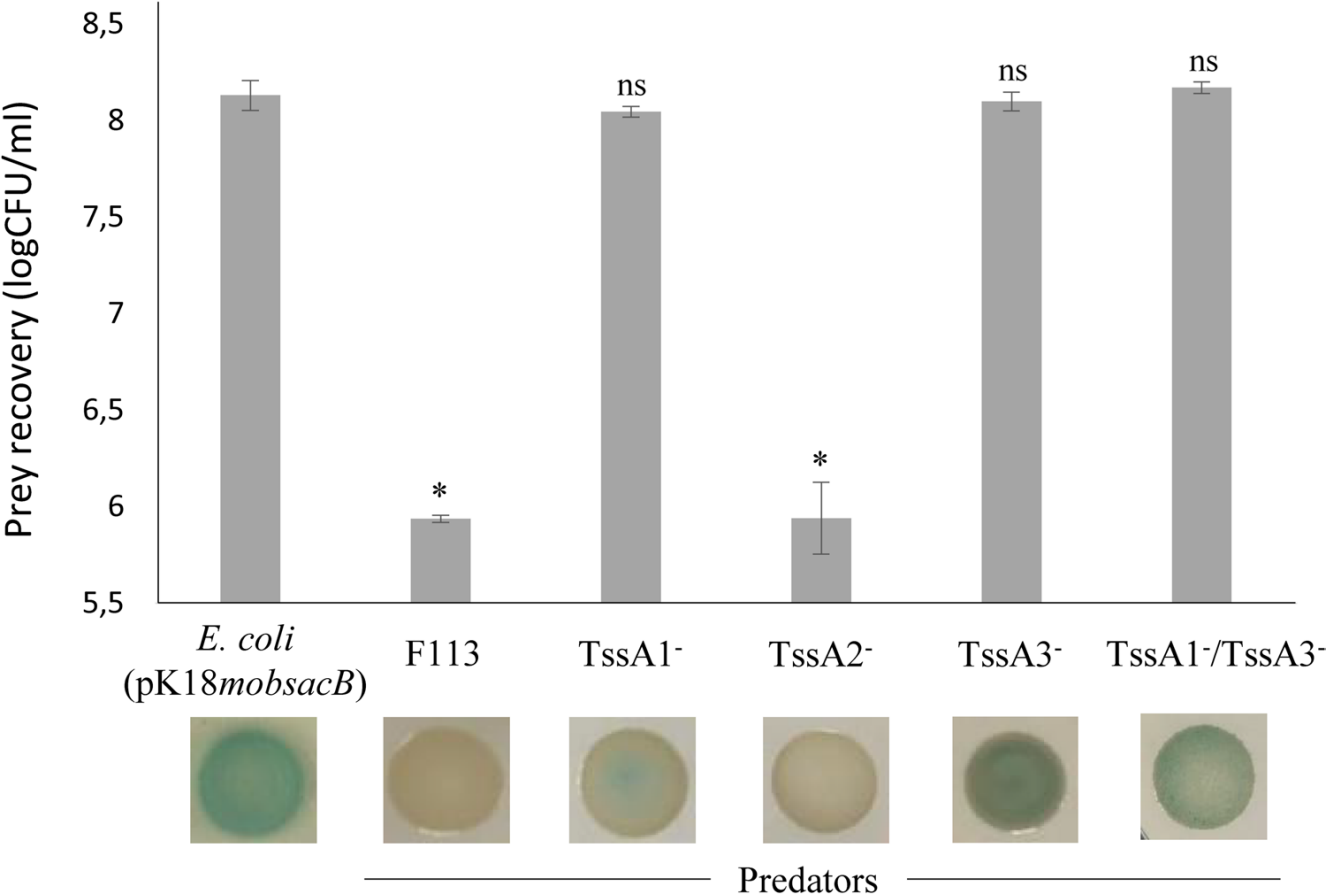
Bacterial killing ability of the three T6SSs of *P. fluorescens* F113. Interbacterial Competition Assay of *P. fluorescens* F113 and T6SS mutants, against an *E. coli* strain carrying pK18*mobsacB* expressing *lacZ*. The bars indicated the amount of prey recovery from each attacker expressed as logCFU/ml. Bottom row shows the *E. coli* growth for each competition experiment. Blue colour in bacterial patches on LB plates supplemented with X-gal and kanamycin indicates *E. coli* growth. Error bars indicate the mean ± s.d. of three biological replicates, and significance was calculated using ANOVA test (*P < 0.01); ns, not significant differences when compared to non-competing *E. coli*.

The *tssA* genes of *P. fluorescens* F113 were selected as targets for the construction of insertional mutants. Mutants affected in each of the three systems were used as predators: *tssA1*^−^, *tssA2*^−^ and *tssA3*^−^ for F1-, F2- and F3-T6SS, respectively. Additionally, a double mutant *tssA1*^−^/*tssA3*^−^ was constructed and used as predator. In the bacterial competition assays (Fig. 5), the wild-type strain was able to kill *E. coli* cells efficiently as observed for the significant prey survival reduction. However, single and double mutants in the structural genes *tssA1* and *tssA3* (F1- and F3-T6SSs) were affected in *E. coli* killing (Fig. 5), showing a survival rate of the prey similar to the control without predator. These results indicate that both systems are functional and have bactericidal activity, as has been shown before for other T6SSs in pseudomonads^4,90,91^. By contrast, the *tssA2* mutant (F2-T6SS) exhibited the same capacity to outcompete *E. coli* that the wild type strain, indicating that the F2-T6SS is not involved in the antibacterial activity of *P. fluorescens* F113 against *E. coli* under our experimental conditions. It is interesting to note that we have not observed expression of the genes encoding this system in any of the tested conditions, suggesting that the lack of function might be a consequence of lack of expression and therefore, the F2-T6SS could be functional under other yet-unknown conditions. Lack of expression and/or antibacterial activity has been observed in other pseudomonads in laboratory conditions, like *P. aeruginosa* wild type strain^2,52^ and *P. putida* K2- and K3-T6SS^38^.

### *Pseudomonas fluorescens* F113 T6SSs are important for the adaption to the rhizosphere microbiome

**Figure 6:**
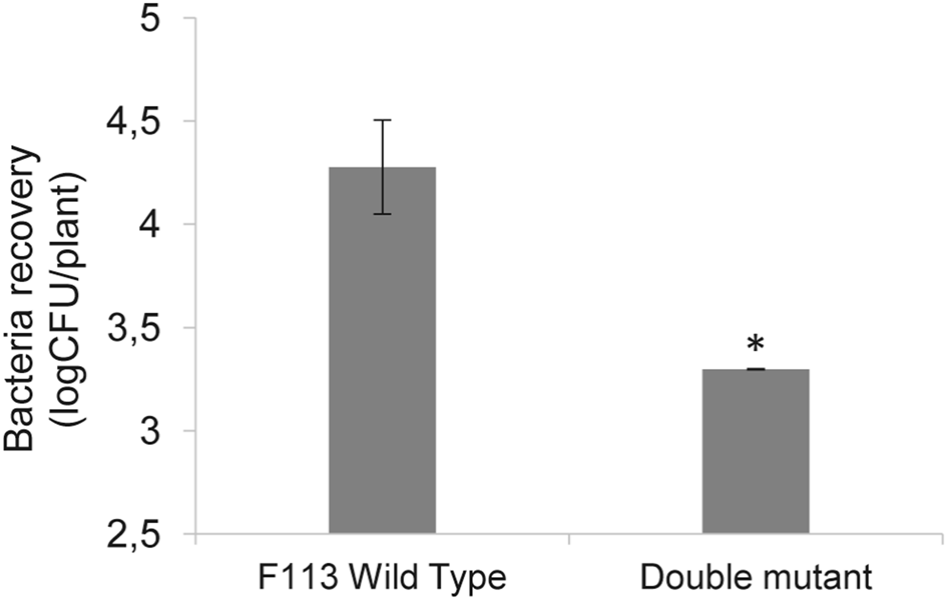
Quantification of recovered cells from rhizosphere colonization. *P. fluorescens* F113 and the derivative strain TssA1^-^/A3^-^ double mutant were inoculated in the rhizosphere of tomato plants grown in agricultural non-sterile soil. Error bars indicate the mean ± s.d. of biological replicates, significance was calculated using Kruskal–Wallis non-parametric test (*P < 0.01).

It has been described that the T6SSs have a role in modulating and shaping the natural microbiota and, in the case of plant-associated bacteria, these weapons are of interest for bacterial persistence in the plant niche, reviewed in^92^. Also Vacheron et al., 2019, showed that T6SS of *P. protegens* contributed to the invasion of the gut microbiome of an insect. Therefore, we wanted to know whether F1 and F3 T6SS could play a role in F113 competence in the rhizosphere. We inoculated 7-day-old tomato plants growing in agricultural, non-sterile soil microcosms, with the wild type strain and the double mutant F1^−^/F3^−^ strain. Bacteria from microcosms were isolated 2 weeks after inoculation and F113 derivatives were selected by using their rifampicin marker resistance. As shown in Fig. 6, when the inoculation is done with the double mutant strain, there is a significant (90%) reduction in the bacterial recovery after rhizosphere colonization compared with the wild-type strain. This result shows that the two tested T6SSs play a relevant role in invading, establishing and/or persisting in the tomato rhizosphere microbiome. The effect of these T6SSs in microbiome adaption is likely due to their role in the inter-bacterial competition that might confer F113 with the capacity to outcompete foes.

## Conclusions

Three T6SS clusters (F1-, F2- and F3-T6SS) and eight effectors (Tfe1, 2, 3, 4, 5, 6, 7, 8) together with 6 immunity proteins (Tfi1, 2, 3, 4, 6 y 8) have been identified in *P. fluorescens* F113. At least two of these systems, F1- and F3-T6SS are functional in laboratory conditions and more importantly in the rhizosphere and possess bactericidal activity. The systems are crucial elements in the colonization of the rhizosphere, most likely providing by providing F113 with the capacity to fight competitors from the rhizosphere microbiome.

## Material and methods

### Bacterial strains and growth conditions

The bacterial strains and mutant constructions used in this study are listed in Supplementary Table S1. The *E. coli* strains were grown in LB medium^93^ at 37 °C. *Escherichia coli* DH5α cells were used for cloning purposes. *P. fluorescens* strains were grown in Minimal Sucrose-Asparagine medium (SA)^94^ at 28 °C. Kanamycin was added to the medium for selection (50 μg ml^−1^ for *P. fluorescens* F113 and 25 μg ml^−1^ for *E. coli*) and in the bacterial killing experiments.

### Bioinformatic analyses

*Pseudomonas* gene sequences were obtained from the *Pseudomonas* Genome database^95^. BLASTP analyses were performed at the NCBI website^96^ and amino acid sequence searches using SMART^97,98^. The Protein Homology/analogy Recognition Engine (Phyre^2^) server was used to perform structural-base homology prediction^99^. The phylogenetic tree was constructed using MEGA X^100^. PSORTb v 3.0.2 software was used to predict subcellular location of proteins^101^, TMHMM server v.2.0 to predict transmembrane domains^102^, and SignalP to predict signal peptides^103^. The molecular biology tools included in Benchling platform were used to identify novel open reading frames (ORFs) (Benchling, Inc., https://benchling.com/academic) and the putative coding proteins were run using NCBI BLASTP tool to determine the degree of conservation.

### Construction of T6SS mutants

Insertional single mutants in PSF113_2422 (*tssA3*), 5797 (*tssA1*) and 5829 (*tssA2*) genes were constructed by electroporation of the plasmid vector pCR2.1-TOPO (Invitrogen) carrying an internal region of the corresponding gene (400 bp *ca*.), in the case of the double mutant *tssA1*^*−*^*/A3* the plasmid vector pG18*mob2* was employed. Single and double recombinant mutants were selected by kanamycin (25 μg ml^−1^) and gentamycin (4 μg ml^−1^) resistance in SA medium and checked by PCR and Southern Blot. Primers used are listed in Supplementary Table S2.

### Bacterial RNA isolation from cultures

RNA was isolated from *P. fluorescens* F113 cultures grown in Minimal Sucrose-Asparagine (SA) medium at exponential (OD_600_ = 0.6) and stationary phase (OD_600_ = 1.2) as indicated in^84^.

### Bacterial RNA isolation from the rhizosphere

Rhizosphere colonization of alfalfa (*Medicago sativa* var. Resis) plants was performed essentially as described in^104^. Seven-day-old seedlings growing in Falcon tubes, using vermiculite as substrate, were inoculated with 1 mL (1 × 10^8^ CFU) of *P. fluorescens* F113 or derivatives *amrZ*^-^ and *fleQ*^-^ mutants. RNA was extracted seven days post-inoculation. Aerial parts of alfalfa plants were removed, 4 mL of Phosphate-buffered saline (PBS) and 6 mL RNAlater^105^ added and tubes were vortexed for 2 min to resuspend bacterial cells. The mix of the 32 preparations per sample was filtered through four layers of sterile muslin cloth in pyrex funnels and separated into six 50 mL tubes. The filtrate was centrifuge 1 min at 1000 rpm and 4 °C. The supernatant was transferred to a sterile tube and centrifuged at 10,000 rpm for 20 min and 4 °C. Supernatants were discarded and pelleted cells dried before liquid nitrogen freezing. RNA isolation was performed as indicated in^84^.

### RNA sequencing

Qubit fluorometer quality assessment, rRNA depletion, strand-specific library construction, and sequencing were performed by Novogene Co., Ltd. (Beijing, China, and Cambridge, UK) using Illumina HiSeq paired-end, 2 × 150 bp.

### Bioinformatic RNA-Seq data processing

After sequencing the RNA, the quality of the raw reads was checked using FastQC. Then, sequence reads were clipped and filtered using Trimmomatic v 0.35^106^ to remove chaperones and low-quality nucleotides, defining a four nts sliding window with an average phred quality of 15 and 50 nts as minimum read length. High-quality reads were directly used for transcript-level quantification using Salmon software^107^, which performs a quasi-mapping to the new annotation of the *P. fluorescens* F113 CDSs (GenBank: NC_016830) and transcript quantification. Normalization of counts and differential gene expression was calculated with DESeq2 1.24.0 R package^108^. Differential gene expression comparisons were made setting a threshold for log_2_ fold change (mutant/wild-type) of ≤  − 1/≥ 1 and a stringent p-adjusted value cutoff ≤ 0.001. RNA-Seq reads have been deposited in to the NCBI Sequence Read Archive database and it is available under the BioProject PRJNA419480: BioSamples accessions: F113 culture in exponential growth phase (SAMN17839758 and SAMN17839763), F113 culture in stationary growth phase (SAMN17839759 and SAMN17839764), F113 from the rhizosphere (SAMN17839757 and SAMN17839762), F113 *amrZ* mutant from the rhizosphere (SAMN17839760 and SAMN17839765) and F113 *fleQ* mutant from the rhizosphere (SAMN17839761 and SAMN17839766).

### Interbacterial competition assays

Competition assays to assess T6SSs were performed on solid media plates according to the previously reported protocol^51^. *Pseudomonas fluorescens* F113 or isogenic derivatives (predator) and *E. coli* DH5α containing pK18*mobsacB* (prey) were grown overnight in LB medium. Next morning, each culture was adjusted to OD_600_ of 1.0. 100 μL of predator and 100 μL of prey strains were mixed and co-cultured for 5 h at 200 rpm and 28 °C. 20 μL of each culture was spotted onto LB-agar supplemented with 5-bromo-4-chloro-3-indolyl-D-galactopyranoside (X-gal) and kanamycin and incubated at 28 °C. Additionally, serial dilutions of the different assays were plated to quantify the number of CFUs in each of them. Three biologically independent experiments were performed.

### Rhizosphere colonization analysis

Tomato seeds (Rebelion F1, Vilmorin, France) were sterilized with 70 % ethanol and 5 % hypochlorite, washed and germinated on sterile 1.0 % agar plates for 24 h at 28 °C. Seedlings (one per tube) were then transferred into sterile 50 mL Falcon tubes containing 16 g of a mix 1:1 of agricultural soil and sterile sand (Merck KGaA, Darmstadt, Germany). Each tube was embedded with 3 mL of autoclaved water, and incubated in a controlled environment room (25 °C, 16 h light cycle). After seven days, each tube/plant was inoculated with 1 mL (1 × 10^6^ CFU/mL) culture of either the *P. fluorescens* F113 wild type strain or the double mutant (*tssA1*^*−*^*/A3*^*−*^) strain. Plants were grown for another two weeks, after which aerial parts were removed; 10 mL of saline solution (0.85 %) was added to each tube and vortexed thoroughly to resuspend the bacteria. After decantation of the soil and sand matrix, serial dilutions of the supernatant were plated onto SA plates supplemented with rifampicin (100 μg mL^−1^) and nystatin (50 μg mL^−1^). Assays were performed with six plants per condition tested and non-inoculated plants were used as a negative control.

### Statistical analyses

The normal distribution of data was checked with Shapiro–Wilk test. When distribution was normal, the data were analysed with one-way ANOVA test, where multiple pairwise-comparisons between strains were performed with Tukey HSD test. When normality was not met, data were analysed with the Kruskal–Wallis non-parametric test. All data were analysed with R package version 3.6.3^109^.

## Supplementary Information

Supplementary Information.
