# Supplementary material for: Pseudomonas fluorescens F113 type VI secretion systems mediate bacterial killing and adaption to the rhizosphere microbiome
Source: Sci Rep. 2021 Mar 11;11:5772. doi: 10.1038/s41598-021-85218-1 (PMC7970981; doi:10.1038/s41598-021-85218-1)
Supplement: Supplementary file 1 — Supplementary Information. [file 41598_2021_85218_MOESM1_ESM.pdf]

Pseudomonas fluorescens F113 type VI Secretion Systems mediate bacterial killing and adaption to the rhizosphere microbiome.

David Durán[1], Patricia Bernal[1,2], David Vazquez-Arias[1], Esther Blanco-Romero[1], Daniel Garrido-Sanz[1], Miguel Redondo-Nieto[1], Rafael Rivilla[1], and Marta Martín[1*]

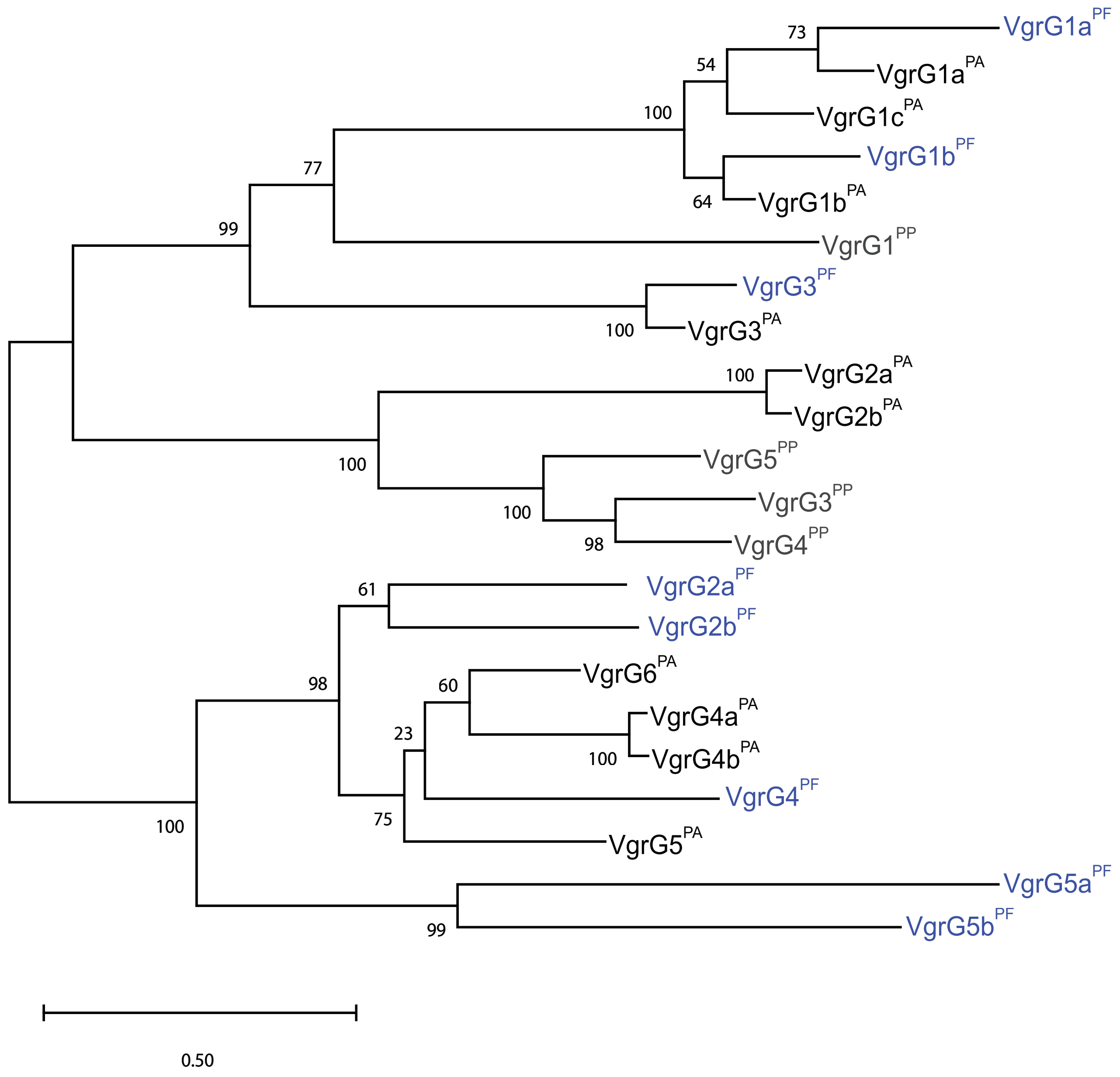

Figure S1. Phylogenetic study of the eight VgrG proteins presents in *P. fluorescens* F113. The VgrG proteins study in this work are indicated in blue. PF; *P. fluorescens*, PA; *P. aeruginosa*, PP; *P. protegens*.

Table S1

The bacterial strains and plasmids employed in this study

**Strains**	**Genotype**	**Source or Reference**
P. fluorescens		
F113	wild type strain, Rif^R	(Shanahan et al., 1992)
tssA₃$^-$	PSF113_2422:: pCR2.1-TOPO, Rif^R Km^R	This work
tssA₁$^-$	PSF113_5797:: pCR2.1-TOPO, Rif^R Km^R	This work
tssA₂$^-$	PSF113_5823:: pCR2.1-TOPO, Rif^R Km^R	This work
$tssA_1^-$ / $tssA_3^-$	PSF113_2422:: pCR2.1-TOPO, Rif^R Km^R, PSF113_5797:: pG18*mob2,* Gm^r	This work
amrZ$^-$	PSF113_4470:: pK19*mobsacB,* Km^R	(Martinez-Granero et al., 2012)
fleQ$^-$	PSF113_1559:: pK18*mobsacB,* Km^R	(Blanco-Romero et al., 2018)
E. coli		
DH5α	*recA1 endA1 gyrA96 thi hsdRl7 supE44 relA1* Δ(*lacZYA-argF*)*U169* (Φ80*lacZ*ΔM15) *deoR phoA*	(Hanahan 1983) Gibco-BRL
Plasmids		
pCR2.1-TOPO	PCR product cloning vector; Ap^r, Km^r	Invitrogen
pK18*mobsacB*	Integrative vector pUC18 derivative; *lacZ mob* site *sacB,* Km^r	(Schafer et al., 1994)
pG18*mob2*	Integrative vector pUC18 derivative *lacZ mob* site *sacB,* Gm^r	(Kirchner and Tauch 2003)

Blanco-Romero, E., M. Redondo-Nieto, F. Martinez-Granero, D. Garrido-Sanz, M. I. Ramos-Gonzalez, M. Martin and R. Rivilla (2018). "Genome-wide analysis of the FleQ direct regulon in Pseudomonas fluorescens F113 and Pseudomonas putida KT2440." Sci Rep **8**(1): 13145.
Hanahan, D. (1983). "Studies on transformation of Escherichia coli with plasmids." J Mol Biol **166**(4): 557-580.
Kirchner, O. and A. Tauch (2003). "Tools for genetic engineering in the amino acid-producing bacterium Corynebacterium glutamicum." J Biotechnol **104**(1-3): 287-299.
Martinez-Granero, F., A. Navazo, E. Barahona, M. Redondo-Nieto, R. Rivilla and M. Martin (2012). "The Gac-Rsm and SadB signal transduction pathways converge on AlgU to downregulate motility in Pseudomonas fluorescens." PLoS One **7**(2): e31765.
Schafer, A., A. Tauch, W. Jager, J. Kalinowski, G. Thierbach and A. Puhler (1994). "Small mobilizable multi-purpose cloning vectors derived from the Escherichia coli plasmids pK18 and pK19: selection of defined deletions in the chromosome of Corynebacterium glutamicum." Gene **145**(1): 69-73.
Shanahan, P., J. O'Sullivan D, P. Simpson, J. D. Glennon and F. O'Gara (1992). "Isolation of 2,4-diacetylphloroglucinol from a fluorescent pseudomonad and investigation of physiological parameters influencing its production." Appl Environ Microbiol **58**(1): 353-358.

Table S2. Primers used in this work

Name	Sequence (5’-3’)	Gene
2422_int_F	TGATCCCCCTGGAGTGGTTG	PSF113_2422 *tssA3*
2422_int_R	TAGGCCTCTTCACGATTGGC	
2422_com_F	GTGAGCTTGCCGATATCGCC	
2422_com_R	TTAGAGAACGCCCAATAACG	
5797_int_F	GGCCTGACGTCCGATATCAC	PSF113_5797 *tssA1*
5797_int_R	GTTGTTGATTTCGCCGCTGA	
5797_com_F	GTGGATGTGCCTTTGTTGCT	
5797_com_R	TTATTCGCTGTCTGGGCCGC	
5829_int_F	CGCCTCAACACGATGATCCA	PSF113_5829 *tssA2*
5829_int_R	TGCCAGCGCAATTCGATAAC	
5829_com_F	ATGTCGTATTCCGGAAAACT	
5829_com_R	CTATTCAATGACCCGTTCCA	

**Table S3:** Distribution of T6SS loci in *Pseudomonas fluorescens strains*									
P. fluorescens strains	Phylogenetic tree code	T6SS clusters	Group 1.1	Group 1.2	Group 2	Group 3	Group 4A	Group 4B	Group 5
Pseudomonas fluorescens 11293									
Pseudomonas fluorescens 2-79									
Pseudomonas fluorescens 74475									
Pseudomonas fluorescens 74954									
Pseudomonas fluorescens 80446/1									
Pseudomonas fluorescens A3422A									
Pseudomonas fluorescens A506	PflA506	2	1			1			
Pseudomonas fluorescens AG1429									
Pseudomonas fluorescens AH4									
Pseudomonas fluorescens AHK-1									
Pseudomonas fluorescens ATCC 13525 - Assembly GCF_900215245.1	CPH89	3	2			1			
Pseudomonas fluorescens ATCC 13525 - Assembly GCF_900215245.1 - Assembly									
Pseudomonas fluorescens ATCC 17400									
Pseudomonas fluorescens AU10414									
Pseudomonas fluorescens AU10973									
Pseudomonas fluorescens AU11114									
Pseudomonas fluorescens AU11122									
Pseudomonas fluorescens AU11136									
Pseudomonas fluorescens AU11164									
Pseudomonas fluorescens AU11235									
Pseudomonas fluorescens AU11518									
Pseudomonas fluorescens AU11706									
Pseudomonas fluorescens AU12271									
Pseudomonas fluorescens AU12597									
Pseudomonas fluorescens AU12644									
Pseudomonas fluorescens AU13852									
Pseudomonas fluorescens AU14440									

Pseudomonas fluorescens AU14705									
Pseudomonas fluorescens AU14917									
Pseudomonas fluorescens AU20219									
Pseudomonas fluorescens AU2390									
Pseudomonas fluorescens AU2989									
Pseudomonas fluorescens AU5633									
Pseudomonas fluorescens AU6026									
Pseudomonas fluorescens AU6308									
Pseudomonas fluorescens AU7350									
Pseudomonas fluorescens BBc6R8									
Pseudomonas fluorescens BRIP34879									
Pseudomonas fluorescens BS2									
Pseudomonas fluorescens BW11P2									
Pseudomonas fluorescens BWKM6									
Pseudomonas fluorescens C1									
Pseudomonas fluorescens C2									
Pseudomonas fluorescens C3									
Pseudomonas fluorescens C8									
Pseudomonas fluorescens CH229									
Pseudomonas fluorescens CH267									
Pseudomonas fluorescens DSM 50090									
Pseudomonas fluorescens DSM 8569									
Pseudomonas fluorescens E24									
Pseudomonas fluorescens EC1									
Pseudomonas fluorescens EGD-AQ6									
Pseudomonas fluorescens EK007-7t-asp									
Pseudomonas fluorescens EK007-RG4									
Pseudomonas fluorescens et76									
Pseudomonas fluorescens F113		3	1			1	1		
Pseudomonas fluorescens FH5									
Pseudomonas fluorescens FR1	C1C98	3	1			1	1		
Pseudomonas fluorescens FW300-N1B4									
Pseudomonas fluorescens FW300-N2C3	AO356	3	1			1	1		

Pseudomonas fluorescens FW300-N2E2	TK06	3	1			1	1		
Pseudomonas fluorescens FW300-N2E3	AO353	3	1			1	1		
Pseudomonas fluorescens G2Y									
Pseudomonas fluorescens GcM5-1A									
Pseudomonas fluorescens GW456-L13									
Pseudomonas fluorescens H14									
Pseudomonas fluorescens H16									
Pseudomonas fluorescens H21									
Pseudomonas fluorescens H24									
Pseudomonas fluorescens HK44									
Pseudomonas fluorescens HKI0770									
Pseudomonas fluorescens HPB									
Pseudomonas fluorescens ICMP 11288									
Pseudomonas fluorescens ICMP3636									
Pseudomonas fluorescens isolate SRM1									
Pseudomonas fluorescens ITEM 17298									
Pseudomonas fluorescens KENGFT3	AYK59	2	1			1			
Pseudomonas fluorescens L111	A7319	2	1			1			
Pseudomonas fluorescens L228	A7318	2	1			1			
Pseudomonas fluorescens L321	A7317	2	1			1			
Pseudomonas fluorescens LBUM223	VO64	3	1			1		1	
Pseudomonas fluorescens LBUM636	AK972	2	1			1			
Pseudomonas fluorescens LMG 5329									
Pseudomonas fluorescens LY3									
Pseudomonas fluorescens MEP34									
Pseudomonas fluorescens ML11A									
Pseudomonas fluorescens MS82	DBV33								
Pseudomonas fluorescens MYb12									
Pseudomonas fluorescens MYb17									
Pseudomonas fluorescens MYb22									
Pseudomonas fluorescens NBRC 14160									
Pseudomonas fluorescens NCIMB 11764	B723								
Pseudomonas fluorescens NCTC10038	DQO01	3	2			1			

Pseudomonas fluorescens NCTC10392									
Pseudomonas fluorescens NCTC3756									
Pseudomonas fluorescens NEP1	CFN16	2	1				1		
Pseudomonas fluorescens NT0133									
Pseudomonas fluorescens NZ007									
Pseudomonas fluorescens NZ011									
Pseudomonas fluorescens NZ052									
Pseudomonas fluorescens P5A									
Pseudomonas fluorescens PA3G8									
Pseudomonas fluorescens PA4C2									
Pseudomonas fluorescens PCL1751	PF1751	2	1			1			
Pseudomonas fluorescens Pf0-1	Pfl01	2	1				1		
Pseudomonas fluorescens Pf29Arp									
Pseudomonas fluorescens PfR 37									
Pseudomonas fluorescens PICF7	PFLUOLIPICF7	2	1			1			
Pseudomonas fluorescens PL5.4									
Pseudomonas fluorescens Ps_20									
Pseudomonas fluorescens Ps_22									
Pseudomonas fluorescens Ps_40									
Pseudomonas fluorescens Ps_77									
Pseudomonas fluorescens Ps006									
Pseudomonas fluorescens Pt14	BH711	2	1			1			
Pseudomonas fluorescens Q2-87	PflQ2	3	1			1	1		
Pseudomonas fluorescens Q8r1-96	PflQ8	2				1	1		
Pseudomonas fluorescens R124	I1A	2	1				1		
Pseudomonas fluorescens S12									
Pseudomonas fluorescens S613									
Pseudomonas fluorescens SBW25	PFLU	2	1			1			
Pseudomonas fluorescens SC1									
Pseudomonas fluorescens SF39a									
Pseudomonas fluorescens SF4c									
Pseudomonas fluorescens SS101	PflSS101	2	1			1			
Pseudomonas fluorescens TDH40									

Pseudomonas fluorescens TDH5									
Pseudomonas fluorescens TR3									
Pseudomonas fluorescens UK4	HZ99	1		1					
Pseudomonas fluorescens UM270									
Pseudomonas fluorescens Wayne1 - Assembly GCF_000285355.1									
Pseudomonas fluorescens Wayne1 - Assembly GCF_000285955.1									
Pseudomonas fluorescens WH6	PFWH6	2	1			1			
Pseudomonas fluorescens Wood1R									
	27	60							

Table S4. Characteristics of proteins encoded by the *P. fluorescens* F113 F1-T6SS cluster.

Locus name	Protein	Identities with H1-T6SS PAO1/Other identities	Conserved Domains COG/pfam/TIGR (Short Name)/Phyre (P)[a]	Molecular weight (KDa)/pI[b]	Predicted cellular location[c]	Transmembrane Helices[d]	Predicted signal peptide[e]
PSF113_5785	TagQ1	PA0070 (TagQ1) 156/298 50.34%	pfam13488 (Gly-zipper_Omp)	312 a.a. 33/9.6	Unknown	0	Yes
PSF113_5786	TagR1	PA0071 (TagR1) 432/569 75.92%	pfam03781 (FGE-sulfatase)	575 a.a. 63/5.5	Outer membrane	0	Yes
PSF113_5787	TagS1	PA0072 238/399 59.65%	COG4591(LolE)/ pfam02687 (FtsX)	398 a.a. 42.3/8.05	Cytoplasmic Membrane	4	No
PSF113_5788	TagT1	PA0073 (TagT1) 152/236 64.41%	COG1136 (LolD)/ pfam00005 (ABC trans)	238 a.a. 25.4/8.84	Cytoplasmic Membrane	0	No
PSF113_5789	PpkA	PA0074 (PpkA) 702/1053 66.67%	pfam00069 (protein kinase) + cd00198 (vWFA)	1036 a.a. 111.3/6.4	Cytoplasmic Membrane	0	No
PSF113_5790	PppA	PA0075 (PppA) 148/222 66.67%	pfam00481 (PP2C)	225 a.a. 24.1/4.3	Cytoplasmic	0	No
PSF113_5791	TagF	PA0076 (TagF1) 130/218 60%	pfam09867 (DUF2094)/ TIGR03373 (VI_minor_4)	217 a.a. 23.5/4.3	Unknown	0	No
PSF113_5792	TssM1	PA0077 (TssM1) 966/1101 78%	COG3523/pfam14331 (IcmF)/TIGR03348 (VI_IcmF)	1166 a.a. 129/7.8	Cytoplasmic Membrane	2	No
PSF113_5793	TssL1	PA0078 (TssL1) 331/433 76.44%	COG3455/pfam09850 (DotU)/ TIGR03349 (IV_VI_DotU) + cd07185 (OmpA_C-like)	436 a.a. 47.8/8.33	Cytoplasmic Membrane	1	No
PSF113_5794	TssK1	PA0079 (TssK1) 111/328 76.35%	COG3522/pfam05936 (T6SS_VasE)/TIGR03 353 (VI_chp_4)	444 a.a. 48.5/6	Cytoplasmic	0	No
PSF113_5795	TssJ1	PA0080 (TssJ1) 60/143 42%	COG3521/pfam12790 (T6SS-SciN)/TIGR03352 (VI_chp_3)	168 a.a. 18.3/4.8	Unknown	0	No
PSF113_5796	Fha1	PA0081 (FhA1) 283/489 57.9%	COG3456/ pfam00498 (FHA)/ TIGR03354 (VI_FHA)	476 a.a. 51.8/4.7	Cytoplasmic	0	No
PSF113_5797	TssA1	PA0082 (TssA1) 198/345 57.4%	COG3515/pfam06812 (ImpA_N) + TIGR03363 (VI_chp_8)	345 a.a. 37.4/4.3	Cytoplasmic	0	No
PSF113_5798	TssB1	PA0083 (TssB1) 154/164 93.9%	COG3516/pfam05591 (T6SS_VipA)/TIGR03 358 (VI_chp_5)	168 a.a. 18.5/5	Cytoplasmic	0	No
PSF113_5799	TssC1	PA0084 (TssC1) 453/499 90.8%	COG3517/pfam05943 (T6SS_VipB)/TIGR03 355 (VI_chp_2)	500 a.a. 55.9/5.2	Cytoplasmic	0	No
PSF113_5800	TssD1/ Hcp1	PA0085 (Hcp1) 127/161 78.9%	COG3157 (Hcp)/ pfam05638 (T6SS_Hcp)/ TIGR03344 (VI_effect_Hcp1)	162 a.a. 17.5/7	Extracellular	0	No
PSF113_5801	Tfe1	STM0277 (Tae4) 25/110 22.73%	Pfam14113 (Tae4)/	149 a.a. 16.6/9.2	Unknown	0	No
PSF113_5802	Tfi1			145 a.a. 16/5.9	Unknown	0	Yes
PSF113_5803	TssE1	PA0087 (TssE1) 111/168 66%	COG3518/Pfam04965 (GPW_gp25)/TIGR03 357 (VI_zyme)	170 a.a. 18.7/6.4	Unknown	0	No
PSF113_5804	TssF1	PA0088 (TssF1) 503/619 81.3%	COG3519/pfam05947 (T6SS_TssF)/TIGR03 359 (VI_chp_6)	619 a.a. 69.3/7.6	Cytoplasmic	0	No
PSF113_5805	TssG1	PA0089 (TssG1) 235/332 70.8%	COG3520/pfam06996 (T6SS_TssG)/TIGR03 347 (VI_chp_1)	332 a.a. 69.3/7.6	Cytoplasmic	0	No
PSF113_5806	TssH1/ ClpV1	PA0090 (ClpV1) 785/901 87.1%	COG0542 (ClpA) + pfam07724 (AAA_2)/ TIGR03345 (VI_ClpV1)	896 a.a. 98.3/5.1	Cytoplasmic	0	No

PSF113_5807	TssI1/ VgrG1 a	PA0091 (VgrG1) 462/639 72.3%	COG3501 (VgrG)/pfam06715 (Gp5_C) + pfam05954 (Phage_GPD)/TIGR03 361 (VI_Rhs_Vgr)	645 a.a. 72.3/5.7	Cytoplasmic	0	No
PSF113_5808	EagR	PA0094 21/83 25.3%	COG5435/ pfam08786 (DUF1795)	144 a.a. 16.1/5.3	Cytoplasmic	0	No
PSF113_5809	Tfe2		pfam05488 (PAAR_motif) + COG3209 (RhsA)/ TIGR03696 (Rhs_assc_core) + pfam08808 (RES)	1479 a.a. 164.4/6.4	Outer membrane	2	No
PSF113_5810	Tfi2		--	92 a.a. 10.6/4.8	Unknown	0	No
PSF113_5811	Tfe3		pfam05488 (PAAR_motif) + COG3209 (RhsA)/ TIGR03696 (Rhs_assc_core) + pfam14414 (HNH/ENDO VII superfamily with conserved WHH)	1472 a.a. 163/5.9	Unknown	2	No
**PSF113_5811.1**	Tfi3		Pfam09346 (SMI1_KNR4)	136 a.a. 15.6/4.2	Cytoplasmic	0	No

a.a.: amino acids. Newly annotated proteins are in bold. [a]Structural-based homology prediction using the Protein Homology/analogy Recognition Engine (Phyre) server (Kelley, et al., 2009). C stands for Confidence. [b] The molecular weight and isoelectric point (pI) are based on prediction by the software ExPASy (http://www.expasy.ch/tools/pi_tool.html). [c] The cellular localization is based on prediction by PSORTb (http://www.psort.org/psortb/index.html). [d] The prediction of transmembrane domains was determined by TMHMN (http://www.cbs.dtu.dk/services/TMHMM/). [e] The prediction of signal peptides was determined by SignalP (http://www.cbs.dtu.dk/services/SignalP/).

Table S5. Characteristics of proteins encoded by the *P. fluorescens* F113 F2-T6SS cluster

Locus name	Protein	Identities with H2-T6SS PAO1/Other identities	Conserved Domains COG/pfam/TIGR (Short Name)/ Phyre[a]	Molecular weight (KDa)/pI[b]	Predicted cellular location[c]	Transmem-brane helices[d]	Predicted signal peptide[e]
PSF113_5815	Stk1	PA1671 (Stk1) 202/310 65.16%%	COG0515 (SPS1)/pfam00069 (Pkinase)/ TIGR03903 (TOMM_kin_cyc)	310 a.a. 34.4/6.6	Cytoplasmic	0	No
PSF113_5816	Stp1	PA1670 (Stp1) 154/236 65.25%%	COG0631 (PTC1)/ pfam00481 (PP2C)/	240 a.a. 26.2/6	Cytoplasmic	0	No
PSF113_5817	TssM2	PA1669 (IcmF2) 767/1175 65.28%	COG3523 (IcmF) pfam06761(IcmF) TIGR03348 (VI_IcmF)	1147 a.a. 130/8.7	Cytoplasmic membrane	3	No
PSF113_5818	TssL2	PA1668 (DotU2) 190/284 66.9%	COG3455/ pfam09850 (DUF2077)/ TIGR03349 (IV_VI_DotU)	287 a.a. 32.1/6.4	Cytoplasmic	1	No
PSF113_5819	TssK2	PA1667 (HsiJ2) 343/443 77.43%	COG3522/pfam05936 (T6SS_VasE)/ TIGR03353 (VI_chp_4)	443 a.a. 49.7/6.5	Cytoplasmic	0	No
PSF113_5820	TssJ2	PA1666 (Lip2) 99/144 68.75%	COG3521/pfam12790 (T6SS-SciN)/TIGR03352 (VI_chp_3)	167 a.a. 18.6/5.3	Unknown	1	Yes
PSF113_5821	Fha2	PA1665 (Fha2) 144/267 53.93%	COG3456/pfam00498 (FHA)/ TIGR03354 (VI_FHA)	283 a.a. 31.5/4.5	Cytoplasmic	0	No
PSF113_5822	Sfa2	PA1663 (Sfa2) 333/502 66.33%	COG3604 (FhlA)7 pfam00158 (sigma54_activat)/TIGR0181 7 (nifA)	505 a.a. 56.1/6.7	Cytoplasmic	0	No
PSF113_5823	TssH2/ ClpV2	PA1662 (ClpV2) 691/875 78.97%	COG0542 (ClpA) + pfam07724 (AAA_2)/ TIGR03345 (VI_ClpV1)	861 a.a. 94/6.2	Cytoplasmic	0	No
PSF113_5824	TssG2	PA1661 (HsiH2) 265/335 79.1%	COG3520/ pfam06996 (T6SS_TssG)/ TIGR03347 (VI_chp_1)	334 a.a. 38.2/6.5	Cytoplasmic	0	No
PSF113_5825	TssF2	PA1660 (HsiG2) 405/526 77%	COG3519/ pfam05947 (T6SS_TssF)/ TIGR03359 (VI_chp_6)	595 a.a. 67.9/6.3	Cytoplasmic	0	No
PSF113_5826	TssE2	PA1659 (HsiF2) 101/131 77.1%	COG3518/ pfam04965 (GPW_gp25)/ TIGR03357 (VI_zyme)	136 a.a. 14.7/8.5	Unknown	0	No
PSF113_5827	TssC2	PA1658 (HsiC2) 449/490 91.6%	COG3517/ pfam05943 (VipB)/ TIGR03355 (VI_chp_2)	491 a.a. 55.8/5.6	Cytoplasmic	0	No
PSF113_5828	TssB2	PA1657 (HsiB2) 138/163 84.7%	COG3516/pfam05591(VipA) /TIGR03358 (VI_chp_5)	167 a.a. 18.6/5	Cytoplasmic	0	No
PSF113_5829	TssA2	PA1656 (HsiA2) 363/518 70.1%	COG3515 pfam06812 (ImpA-rel_N)/TIGR03363 (VI_chp_8) + pfam16989 (T6SS_VasJ)/TIGR03362 (VI_chp_7)	518 a.a. 58.3/6.4	Cytoplasmic	0	No
PSF113_5830	TssI2/ VgrG2a	PA3294(VgrG4a) 372/689 54%	COG3501 (VgrG)/ pfam05954 (Phage_GPD)/ TIGR03361 (VI_Rhs_Vgr)	687 a.a. 77.4/6.7	Cytoplasmic	0	No
PSF113_5831	Tap2	PA3293 (Tap) 21/68 31%	pfam13503 (DUF4123)	284 a.a. 32.7/6.4	Unknown	0	No
PSF113_5832	Tfi4			256 a.a. 28.6/7.5	Unknown	0	No
PSF113_5833	Tfe4		COG3675 (Lip2)/ pfam01764 (Lipase_3)	727 a.a. 81.4/6.4	Cytoplasmic	0	No

a.a.: amino acids. Newly annotated proteins are in bold. [a]Structural-based homology prediction using the Protein Homology/analogy Recognition Engine (Phyre) server (Kelley, et al., 2009). C stands for Confidence. [b] The molecular weight and isoelectric point (pI) are based on prediction by the software ExPASy (http://www.expasy.ch/tools/pi_tool.html). [c] The cellular localization is based on prediction by PSORTb (http://www.psort.org/psortb/index.html). [d] The prediction of transmembrane domains was determined by TMHMN (http://www.cbs.dtu.dk/services/TMHMM/). [e] The prediction of signal peptides was determined by SignalP (http://www.cbs.dtu.dk/services/SignalP/).

Table S6. Characteristics of proteins encoded by the *P. fluorescens* F113 F3-T6SS cluster

Locus name	Protein	Identities with H2-T6SS PAO1/Other identities	Conserved Domains COG/pfam/TIGR (Short Name)/ Phyre[a]	Molecular weight (KDa)/pI[b]	Predicted cellular location[c]	Transmem-brane helices[d]	Predicted signal peptide[e]
PSF113_2422	TssA3	PA2360 (HsiA3) 215/367 59%	COG3515 pfam06812 (ImpA-rel_N)/TIGR03363 (VI_chp_8)	360 a.a. 40.5/4.9	Cytoplasmic	0	No
PSF113_2421	TssM3	PA2361 (IcmF3) 611/1243 49.16%	COG3523 (IcmF) Pfam14331 (IcmF) TIGR03348 (VI_IcmF)	1274 a.a. 142.6/5.6	Cytoplasmic membrane	2	No
PSF113_2420	TssL3	PA2362 (DotU3) 149/248 60%	COG3455/ pfam09850 (DotU)/ TIGR03349 (IV_VI_DotU)	254 a.a. 28.4/6.1	Cytoplasmic membrane	1	No
PSF113_2419	TssK3	PA2363 (HsiJ3) 309/442 70%	COG3522/pfam05936 (T6SS_VasE)/ TIGR03353 (VI_chp_4)	443 a.a. 48.2/5	Cytoplasmic membrane	0	No
PSF113_2418	TssJ3	PA2364 (Lip3) 93/134 69.4%	COG3521/pfam12790 (T6SS-SciN)/TIGR03352 (VI_chp_3)	149 a.a. 18.1/8.9	Unknown	1	Yes
PSF113_2417	TssB3	PA2365 (HsiB3) 137/159 86.2%	COG3516/pfam05591(VipA)/TIGR03358 (VI_chp_5)	186 a.a. 20.9/4.6	Cytoplasmic	0	No
PSF113_2416	TssC3	PA2366 (HsiC3) 456/494 92.3%	COG3517/ pfam05943 (VipB)/ TIGR03355 (VI_chp_2)	493 a.a. 55/5.6	Cytoplasmic	0	No
PSF113_2415	TssD3/ Hcp3	PA2367 (Hcp3) 143/165 86.7%	COG3157 (Hcp)/ pfam05638 (T6SS_HCP)/ TIGR03344 (VI_effect_Hcp1)	165 a.a. 17.6/4.7	Extracellular	0	No
PSF113_2414	TssE3	PA2368 (HsiF3) 82/143 57.3%	COG3518/ pfam04965 (GPW_gp25)/ TIGR03357 (VI_zyme)	147 a.a. 16.7/6.5	Cytoplasmic	0	No
PSF113_2413	TssF3	PA2369 (HsiG3) 460/597 77%	COG3519/ pfam05947 (T6SS_TssF)/ TIGR03359 (VI_chp_6)	596 a.a. 65.9/6.2	Cytoplasmic	0	No
PSF113_2412	TssG3	PA2370 (HsiH3) 239/341 70.1%	COG3520/ pfam06996 (T6SS_TssG)/ TIGR03347 (VI_chp_1)	341 a.a. 37.4/9.6	Unknown	0	No
PSF113_2411	TssH3/ ClpV3	PA2371 (ClpV3) 724/849 85.3%	COG0542 (ClpA) / TIGR03345 (VI_ClpV1) + pfam07724 (AAA_2)	847 a.a. 92.4/5.5	Cytoplasmic	0	No
PSF113_2410			P: Coronavirus RNA-binding domain a.a. 121-173 C: 80.5	188 a.a 21.4/6.9	Unknown	0	No
PSF113_2409	TssI3/ VgrG3	PA2373 (VgrG3) 547/669 81.8%	COG3501 (VgrG)/ pfam05954 (Phage_GPD) + pfam06715 (Gp5)/ TIGR03361 (VI_Rhs_Vgr)	669 a.a. 72.5/5.6	Cytoplasmic	0	No
PSF113_2408	Tfe5	PA2374 (TseF) 117/176 66.5%	COG2849 (YwqK)	180 a.a. 20/6.9	Unknown	0	No
PSF113_2407	paar-like	PA2375 110/131 84%	pfam14107 (DUF4280)	131 a.a. 13.2/7	Cytoplasmic membrane	0	No
PSF113_2406	Kinase (non-T6 related	PA1829 296/356 83.15%	COG3173 (YcbJ) / pfam01636 (APH) / TIGR02721 (ycfN_thiK)	355 a.a. 40.8/5.4	Cytoplasmic	0	No
PSF113_2405	Short-chain dehydrogenase (non-T6 related)	PA1828 232/255 91%	COG1028 (FabG)/ pfam13561 (adh_short_C2) /TIGR01838 (3oxo_ACP_reduc)	255 a.a. 26.7/7.9	Cytoplasmic	0	No
PSF113_2404	Oxidoreductase (non-T6 related)	PA0237 92/295 31.2%	COG0673 (MviM) / pfam01408 (GFO_IDH_MocA) / TIGR04380 (myo_inos_iolG)	371 a.a. 40.6/6.8	Periplasmic	0	No

PSF113_2403	SpvB + TcdB		pfam03534 (SpvB) + pfam12255 (TcdB_toxin_midC) + pfam12256 (TcdB_toxin_midN)	1434 a.a. 162/4.8	Unknown	0	No
PSF113_2402	RhS + Cter (TccC)	PSF113_ 2401- 2402 552/667 83%	COG3209 (RhsA) / TIG03696 (Rhs_assc_core) + PRK15386	911 a.a. 103/5.3	Unknown	0	No
PSF113_2401	RhS + Cter (TccC)		COG3209 (RhsA) / TIG03696 (Rhs_assc_core)	945 a.a. 106/5.5	Cytoplasmic	0	No

a.a.: amino acids. Newly annotated proteins are in bold. [a]Structural-based homology prediction using the Protein Homology/analogy Recognition Engine (Phyre) server (Kelley, et al., 2009). C stands for Confidence. [b] The molecular weight and isoelectric point (pI) are based on prediction by the software ExPASy (http://www.expasy.ch/tools/pi_tool.html). [c] The cellular localization is based on prediction by PSORTb (http://www.psort.org/psortb/index.html). [d] The prediction of transmembrane domains was determined by TMHMN (http://www.cbs.dtu.dk/services/TMHMM/). [e] The prediction of signal peptides was determined by SignalP (http://www.cbs.dtu.dk/services/SignalP/).

Table S7. Characteristics of proteins encoded by orphan *hcp* and *vgrG* gene clusters

Locus name	Gene name Protein name	Identities	Conserved Domains COG/pfam/TIGR (Short Name)/Phyre[a]	Molecular weight (KDa)/pI[b]	Predicted cellular location[c]	Transmembrane helices[d]	Predicted signal peptide[e]	Predicted non-classically secreted protein[f]
**Orphan Hcp2 cluster**								
PSF113_1976.1				49 a.a.	Unknown	0	No	No
PSF113_1976	Hcp2	PA1512 (HcpA) 130/166 (78%)	COG3157 (Hcp)/pfam05638 (T6SS_HCP)/TIGR03344 (VI_effect_Hcp1)	166 a.a. 18.9/5.5	Extracellular	0	No	No
PSF113_1975.1				90 a.a. 9.7/10.5	Extracellular	0	No	Yes
PSF113_1975				80 a.a. 9.1/4	Unknown	0	No	No
**Orphan VgrG1b cluster**								
PSF113_2885	VgrG1b	PA0095 (VgrG1b) 571/741 (77%)	COG3501 (VgrG)/ pfam05954 (Phage_GPD)/+pfam06715 / TIGR03361 (VI_Rhs_Vgr) + pfam10106	741 a.a. 83.5/5.2	Cytoplasmic	0	No	No
PSF113_2886		PA0096 118/152 77.6%	P: Partial gp5 -OB fold (17-83 a.a.) C: 99.4%	153 a.a. 16/6.6	Unknown	0	No	No
PSF113_2887		PA0097 156/366 42.6%	COG5351/pfam09937 (DUF2169)	380 a.a. 42.3/6.3	Cytoplasmic	0	No	Yes
PSF113_2888		PA0098 141/340 41.5%	COG0304 (FabB)/ TIGR01486 (HAD-SF-IIB-MPGP)	351 a.a. 37.3/6.2	Cytoplasmic	0	No	No
PSF113_2888.1	Tfe6	PA0099 80/140 57%	pfam13665 (DUF4150-PAAR-like) + C-term	305 a.a. 32.7/9	Unknown	0	No	Yes
PSF113_2889	Tfi6			246 a.a. 27.6/4.3	Unknown	0	No	No
PSF113_2890		PA0101 252/405 62%	pfam13646 (HEAT2)/TIGR02270	413 a.a. 46.4/5.7	Cytoplasmic	0	No	No
**Orphan VgrG2b cluster**								
PSF113_0495	VgrG2b	PA5266 (VgrG6) 364/615 (59.2%)	COG3501 (VgrG)/ pfam05954 (Phage_GPD)/ TIGR03361 (VI_Rhs_Vgr)	685 a.a. 76/6.7	Cytoplasmic	0	No	No
PSF113_0496	Tap2b		pfam13503 (DUF4123)	202 a.a. 22.7/4.4	Unknown	0	No	No
PSF113_0497	Tfe7		COG2961(RlmJ)/ pfam04378 (RsmJ)	279 a.a. 31.7/7.4	Cytoplasmic	0	No	No
**Orphan VgrG2c cluster**								
PSF113_0666	VgrG5a	PA3486 (VgrG4b) 150/458 (33%)	COG3501 (VgrG)/ pfam05954 (Phage_GPD)/ TIGR03361 (VI_Rhs_Vgr)	435 a.a. 47.2/6.2	Cytoplasmic	0	No	No
PSF113_0667	Tap5a		pfam13503 (DUF4123)	280 a.a. 31.7/8.3	Unknown	0	No	No
PSF113_0668	Tfe8		Pfam01554 (MatE)	449 a.a.	Cytoplasmic membrane	10	No	No
PSF113_0669	Tfi8			97 a.a.	Cytoplasmic membrane	2	No	No
PSF113_0670	Non-T6SS							
PSF113_0671	Non-T6SS							

PSF113_0672	Toxin?		DUF4136					
PSF113_0673	Toxin?		DUF4136					

**Orphan VgrG2d cluster**								
PSF113_3144	vgrG4	PA3294 (VgrG4a) 259/460 56%	COG3501 (VgrG)/ pfam05954 (Phage_GPD)/ TIGR03361 (VI_Rhs_Vgr)	442 a.a. 50.7/6.7	Cytoplasmic	0	No	No
PSF113_3144a		PA4863 54/127 42.5%	COG0456 (RimI)/ pfam13508 (Acetyltransf_7)/ TIGR02406 (ectoine_EctA)	153 a.a. 17/7.2	Cytoplasmic	0	No	No

**Orphan VgrG2e cluster**								
PSF113_3904	vgrG5b	PA3294 (VgrG4a) 181/461 39%	COG3501 (VgrG)/ pfam05954 (Phage_GPD)/ TIGR03361 (VI_Rhs_Vgr)	472 a.a. 52.8/6.8	Cytoplasmic	0	No	No

a.a.: amino acids. Newly annotated proteins are in bold. Partial proteins or those with premature stop codon are underline. [a]Structural-based homology prediction using the Protein Homology/analogy Recognition Engine (Phyre) server (Kelley, et al., 2009). C stands for Confidence. [b]The molecular weight and isoelectric point (pI) are based on prediction by the software ExPASy (http://www.expasy.ch/tools/pi_tool.html). [c]The cellular localization is based on prediction by PSORTb (http://www.psort.org/psortb/index.html). [d]The prediction of transmembrane domains was determined by TMHMN (http://www.cbs.dtu.dk/services/TMHMM/). [e]The prediction of signal peptides was by use of SignalP (http://www.cbs.dtu.dk/services/SignalP/). [f]The prediction was determined by SecretomeP (http://www.cbs.dtu.dk/services/SecretomeP/).
